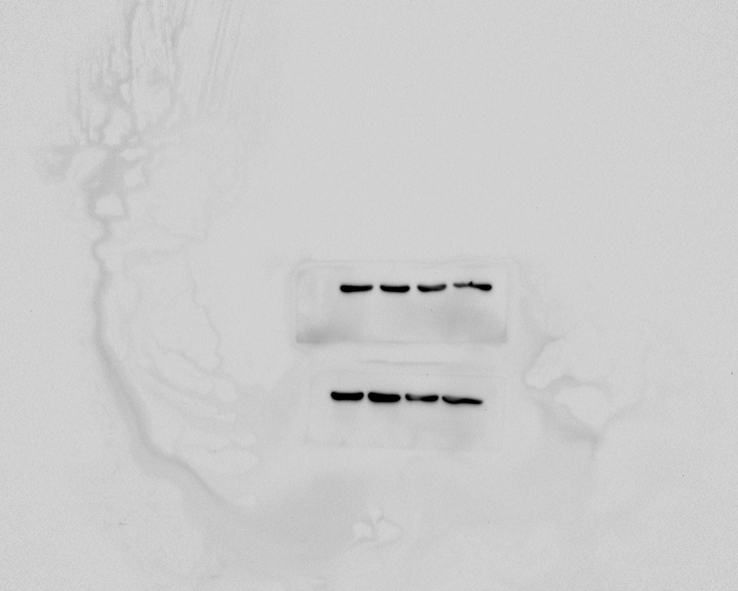# Anti-rheumatoid arthritis potential of different fractions derived from of *Coluria longifolia*

**DOI:** 10.1016/j.heliyon.2023.e23893

**Published:** 2023-12-22

**Authors:** Yan-ping Gao, Qiuting Ma, Jian Liang, Qiang Wu, Yu-ye Zhu, Xi-de Ye, Zhiyong Liu

**Affiliations:** a Research Center for Traditional Chinese Medicine Resources and Ethnic Minority Medicine, Jiangxi University of Chinese Medicine, Nanchang, 330004, China; b Science & Technology Center for Experimental Animal of Jiangxi University of Chinese Medicine, Nanchang, 330004, China; c Nanchang Medical College, Nanchang, 330004, China; d School of Pharmacy, Jiangxi University of Chinese Medicine, Nanchang, 330004, China

**Keywords:** *Coluria longifolia*, Rheumatoid arthritis, Anti-arthritic activity, CIA rat model, Mechanism

## Abstract

*Coluria longifolia* Maxim (*C. longifolia*) is a Chinese folk medication commonly used to treat arthritis and joint pain. Literatures have reported that *C. longifolia* has significant anti-inflammatory, analgesic and antipyretic effects. The aim of this research was to assay the effective fractions of *C. longifolia* (EFCL) against rheumatoid arthritis (RA) and to elucidate its anti-RA mechanism on a preliminary basis. The rat model of collagen-induced arthritis (CIA) was established. The therapeutic effects of different fractions *in vivo* were evaluated by body weight changes, a foot swelling score, inflammatory factors and histopathological examination. The mechanism of EFCL was investigated by activity of oxidative stress related enzyme, qPCR and Western blotting tests. I*n vivo* results showed that total extraction (TE) and *n*-butanol fraction (NF) could significantly alleviate the symptoms of RA, decrease the levels of IL-6 and TNF-α (*P* < 0.01), and improve histopathological injury. The mechanism study showed that SOD level was significantly increased with MDA level decreased in the NF group. The upregulated proteins and mRNA expression levels of Nrf2, HO1 and NQO1 after TE and NF administration suggested that the anti-arthritic effect may be related to the Nrf2 signaling pathway and downstream HO1 and NQO1. In conclusion, this study confirmed that *C. longifolia* is capable of treating RA with NF as the main effective fraction. Its anti-RA action may be associated with Nrf2 signaling pathway and downstream HO1 and NQO1.

## 1 Introduction

Rheumatoid arthritis (RA) is a common clinical chronic, systemic, and recurrent autoimmune disease [1]. Currently, commonly used drugs for the treatment of rheumatoid arthritis (RA) include nonsteroidal anti-inflammatory drugs (NSAIDs), glucocorticoids, disease-modifying antirheumatic drugs (DMARDs), and biologics [2]. However, long-term use of these drugs may lead to gastrointestinal bleeding and kidney damage, as well as side effects such as osteoporosis, hypertension, and diabetes. They may also cause immune suppression and increase the risk of infection [3]. In order to find new RA drugs, researchers are exploring new targets and drug design strategies. Traditional Chinese medicine treatment for RA is often based on the "syndrome," which means using multi-target and multi-pathway Chinese medicine to treat the etiology, pathology, and lesions of RA, and it has shown significant efficacy and safety advantages in most cases [[4], [5], [6]].

*Coluria longifolia* Maxim. (*C. longifolia*), a plant belonging to the genus of *Coluria* (Rosaceae), is unique to China. It is mainly distributed in Gansu, Qinghai, Yunnan and Tibet provinces. It grows at an altitude of 2700 - 4100 m and is commonly used in the treatment of arthritis and joint pain in traditional life [7]. Wei et al. found that *C. longifolia* has significant anti-inflammatory, analgesic and antipyretic effects [8]. In our previous studies, it was found that *C. longifolia* had good anti-rheumatoid arthritis (RA) activity as well, and further in vitro studies showed that the *n*-butanol fraction is the anti-arthritic active fraction [9,10]. Until now, there have been few chemical investigations on *C. longifolia*, only a few triterpenoids, flavonoids and phenylpropanoids were obtained in our preliminary studies [[11], [12], [13], [14]]. As a part of our continuing efforts to find the anti-RA components, the different fractions of *C. longifolia* were investigated in this study, including total extraction (TE), water fractions (WF), *n*-butanol (n-BF), ethyl acetate (EF) and petroleum ether fraction (PF). Collagen-induced arthritis (CIA) rat model was used to screen the *in vivo* effects of different parts on RA. In addition, the activity of RA-related cytokines and oxidative stress-related enzymes was investigated by enzyme-linked immunoassay, and the expressions of related genes and proteins were detected by qPCR and Western blotting, which preliminary revealed the mechanism of its anti-RA effects.

## 2 Materials and methods

### 2.1 Chemicals and reagents

The whole herb (Batch No.: 2018-08-15) was purchased from Chengdu Lotus Pond Chinese Herbal Drug Market and identified as *Coluria longifolia* Maxim. by Prof. Guo-yue Zhong of Jiangxi University of Traditional Chinese Medicine. The whole plant of *C. longifolia* was dried out and then extracted with 80 % EtOH three times. After the extract was mixed with water, petroleum ether, ethyl acetate and n-butanol were added in turn for extraction. The different extracts were concentrated under vacuum to yield TE, PF, EF, NF and WF, respectively.

Bovine CII collagen and incomplete Freund's Adjuvant (IFA) were bought from Chondrex (USA); an ECL chemiluminescence kit was bought from GeneView (USA); 1 ml syringes were purchased from Becton, Dickinson Co., Ltd. (USA); a fast bench centrifuge, a constant temperature mixer, and electric pipettes were all purchased from Eppendorf (Germany); a high-speed homogenizer was purchased from IKA Co. (Germany). Pure water was prepared by Milli-Q ultra-pure water system (Millipore, France). Dexamethasone was purchased from GlpBio (Montclair, CA, USA). Dimethyl sulfoxide (DMSO), glacial acetic acid and sodium chloride injection were purchased from Sinopharm Chemical Reagent Co., Ltd. (Shanghai, China), and rat serum IL-6 and TNF-α enzyme-linked immunosorbent assay (ELISA) kits were purchased from Bender (Germany).

### 2.2 Rat model of CIA

#### 2.2.1 Animals

Male Wistar rats (180 ± 20 g) were purchased from Hunan Slaccas Laboratory Animals Co., Ltd. (License number: SCXK (xiang) 2019-0004, Hunan, China) and housed in the Experiment Animal Center of Jiangxi University of Traditional Chinese Medicine. All rats were fed under SPF conditions (temperature: 23 ± 2 °C; relative humidity: 45 %–60 %; with a day-night cycle of 12 h; and a regular autoclave diet). *Experiments were performed under a project license (No.* JZSYDWLL20201226) granted by experimental animal Ethics Committee of 10.13039/501100012249Jiangxi University of Traditional Chinese Medicine, in compliance with China guidelines for the care and use of animals.

#### 2.2.2 Grouping and administration

To investigate the potential effect of different fractions of *C. longifolia* on RA, 48 rats were divided into the following 8 groups (n = 6) and orally treated with 1) CMC-Na (control), 2) CMC-Na (model), 3) dexamethasone (positive drug, 0.5 mg/kg/d), 4) TE (300 mg/kg/d), 5) PF (100 mg/kg/d), 6) EF (100 mg/kg/d), 7) NF (100 mg/kg/d) and 8) WF (100 mg/kg/d).

#### 2.2.3 Experimental model

Refer to the previous literature [15] to establish the CIA rat model with slight modifications. Simply speaking, 2 mg/mlL bovine type II collagen was dissolved in 0.05 M glacial acetic acid. Then the CII solution was mixed with an equal volume of IFA and completely emulsified. Rats were intradermally injected with CII emulsion at the tail root (200 μg each). One week later, 100 μg CII emulsion was injected subcutaneously into the tail root for secondary immunization. Normal rats of the control group were injected with saline through the tail root. About two weeks later, the immunized rats showed obvious symptoms of RA, such as inflammation, erythema and swelling at their toe joints. Two weeks after the initial immunization, the rats were orally administered with CMC-Na, dexamethasone and different fractions of *C. longifolia*. Rat foot paw volume and body weight were measured every 4 days during the experiment. Arthritis severity was measured on the following order scales: Grade 0 = absence of signs of arthritis; Grade 1 = red and swollen paws; Grade 2 = foot and claw deformity; Grade 3 = joint stiffness in the foot and paw; Grade 4 = maximum swelling and deformity with joint rigidity [16]. Because incidence of anterior palmar inflammation was quite low and the joints were more prone to severe swelling, the sum of the two hindfeet was used to evaluate the pathogenesis of the rats. After 4 weeks of treatment, blood samples were collected using the abdominal aorta method and rats were rapidly sacrificed by severing the head.

#### 2.2.4 Histological and pathological examination

After the rats were sacrificed, the ankle joints, knee joints and surrounding tissues were taken and preserved with 4 % paraformaldehyde for 24 h. Decalcified with 10 % EDTA-2Na for one month, followed by dehydration with ethanol of different concentrations, paraffin embedding, sections (4 μm), HE staining. Observing the histopathological changes in the ankle and knee joints under a microscope and taking pictures.

#### 2.2.5 Determination of serum cytokine levels

After incubation under ice-cold conditions, serum was obtained and centrifuged at 800 g for 15 min.

Isolated serum samples were stored at −80 °C prior to cytokine expression experiments. Serum IL-6 and TNF-α levels were measured using the ELISA kit following the kit instructions.

#### 2.2.6 Western blotting

Following the published method [17], 30 μg of protein extract was prepared and processed for Western blotting analysis. Briefly, the protein on the membrane reacted with the primary antibody at room temperature for 1 h, then washed three times, and then reacted with the secondary antibody for another 1 h. As directed by the Odyssey infrared imaging system (BIO-RAD, Hercules, CA, USA), the blot was washed three times followed by development and exposure.

#### 2.2.7 Quantitative real-time PCR reverse transcription PCR

RNAiso Plus (Takara, China) was employed to isolate total RNA from spleen cells following the manufacturer's guidelines. Total RNA (1 μg) was converted to cDNA via reverse transcription using the PCR Forward Primer, the PCR Reverse Primer, and the PrimeScript™-RT-Enzyme-Mix (Takara) following the instructions for reverse transcription kit (NO. RR047A, Takara). The quantitative real-time PCR (qPCR) step of the qRT-PCR protocol was performed using the cDNA as the template with TB Green Premix ExTaq™ (Takara) following kit instructions for real-time fluorescence quantification (No. RR420A, Takara). The sequences of the qPCR primers are shown in Table 1.

**Table 1:** The specific primers used for amplification.

Gene	Forward 5′-3′	Reverse 5′-3′
Nrf2	CCCAGCACATCCAGACAGAC	TATCCAGGGCAAGCGACTC
GAPDH	AGACAGCCGCATCTTCTTGT	CTTGCCGTGGGTAGAGTCAT
NQO1	AAATGACAAGGGCGAAAGTG	TGGGACAGAAGACCAAGAGG
HO1	GGAGACACGACGGAGTTCAT	GCACATTTTCTGCCACTTTG

#### 2.2.8 1, 1-Diphenyl-2-picrylhydrazyl (DPPH) radical scavenging activity

The DPPH radical scavenging activity was studied in accordance with the reported method [18]. Different fractions in DMSO were mixed with 40 μg/mL a methanol solution containing DPPH radicals. The mixture was shaken vigorously and left still for 30 min in the dark; then the absorbance was measured at 540 nm versus Vitamin E as positive control using a spectrophotometer (LaboMed Inc., Culver City, CA, USA).

#### 2.2.9 Antioxidant activity

The antioxidant activity of different fractions was estimated by a total antioxidant capacity assay kit with the ferric reducing ability of plasma (FRAP) method (S0116, Beyotime, China) [19]. Briefly, the working solution was prepared by mixing tripyridyltriazine (TPTZ) dilution, detection buffer and TPTZ solution in a ratio of 10:1:1 (v:v:v) and incubated at 37 °C. 5 μL calibration solution or sample solution was added to 180 μL working solution, then they were stored for 5 min. The FRAPs of samples were quantified by a linear calibration curve and expressed as mM FeSO_4_ equivalents by measuring absorbance at 593 nm.

#### 2.2.10 Statistical analysis

All data were analyzed using one-way analysis of variance (ANOVA) followed by Dunnett's multiple comparisons tests (between different groups) and expressed as the mean ± standard deviation. GraphPad Prism 7 for Windows (Graphpad Software Inc., USA) was used for statistical analysis. The difference in *P* < 0.05 was statistically significant.

## 3 Results and discussion

### 3.1 Anti-RA effects of different fractions of C. longifolia

CIA is an experimental autoimmune disease that immunizes rodents (such as rats and mice) and non-human primates by type II collagen (CII). After immunization, these animals develop autoimmune polyarthritis with some clinical and histological features associated with RA [20]. As shown in Fig. 1, the rats in the model group began to show symptoms in succession 12 days after the second immunization. On the 15th day, all rats in the model group showed symptoms. The joint swelling of the rats in the model group was the most serious; while the morbidity of the rats in the TE, NF and WF administration groups obviously improved compared with the model group (Fig. 2). During the experiment, the rats in control group had a glossy fur color, normal diet, and the body weight continued to increase (Fig. 3). As for rats in the model group, their hair color was rough and dark, they experienced diarrhea and loss of appetite, and their weight increased quite slowly with arthritis related symptoms such as redness, swelling in the plantar, toe and ankle joints. On the contrary, there was almost no paw swelling in dexamethasone-treated rats.

**Fig. 1:**
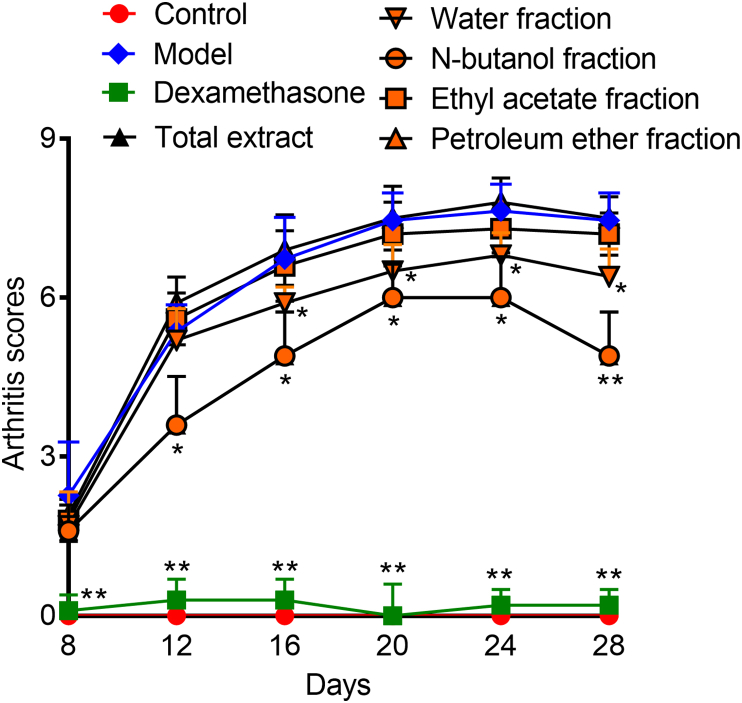
The arthritis scores of CIA rats.

**Fig. 2:**
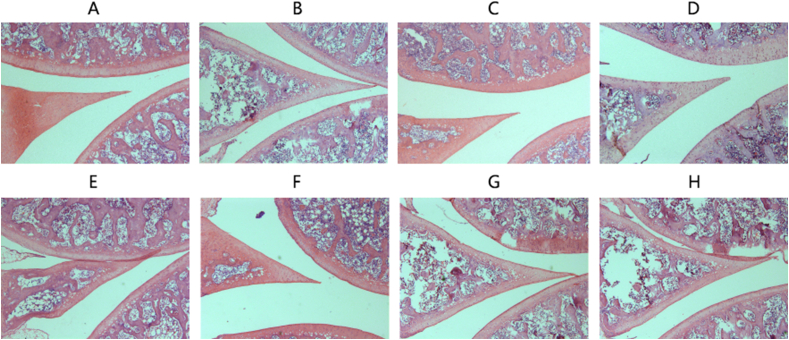
The histopathological examinations of CIA rats.A：control,B:model,C:dexamethasone,D:total extract,E:water fraction,F:n-butanol fraction,G:ethyl acetate fraction,H:petroleum ether fraction.

**Fig. 3:**
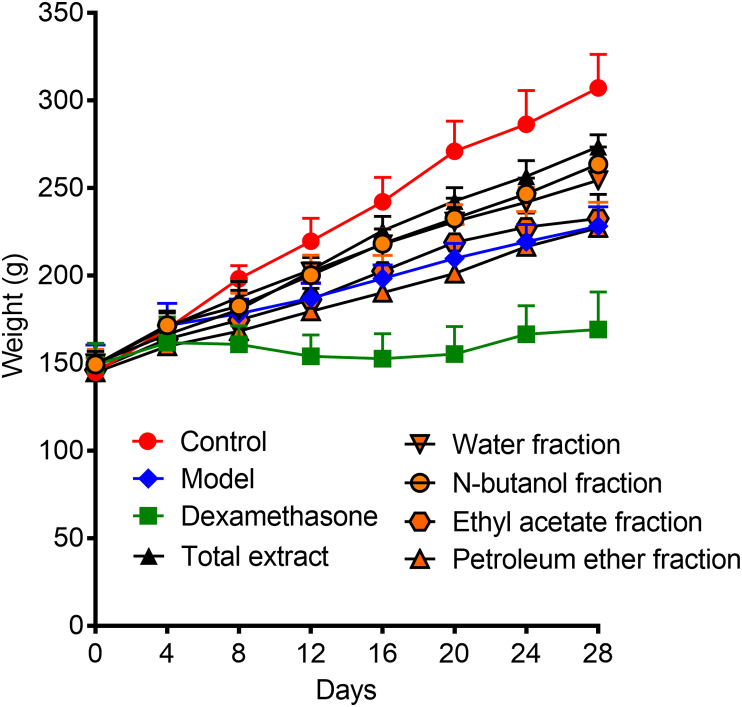
The weight changes of rats.

**Fig. 4:**
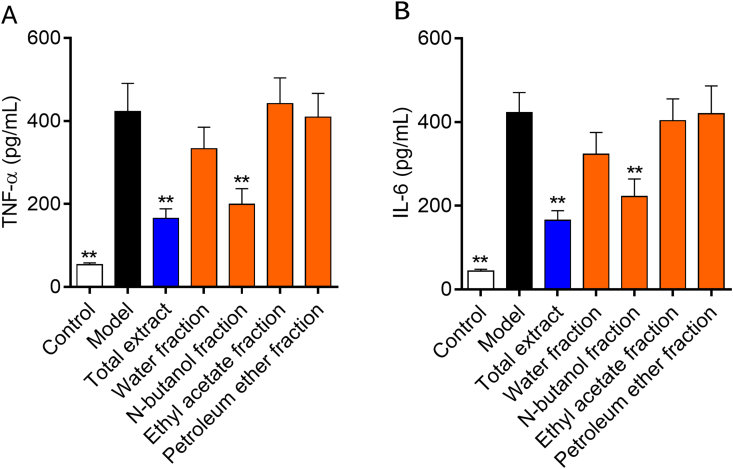
The levels of cytokines of CIA rats. A; TNF-α,B:IL-6.

RA is primarily associated with inflammation and increased levels of pro-inflammatory cytokines [21]. Therefore, serum pro-inflammatory cytokines such as TNF-α and IL-6 were determined on the 28th day after the first immunization. The experimental results showed that the serum levels of pro-inflammatory cytokines in rats of the model group were significantly increased, indicating that the production of pro-inflammatory cytokines were closely related to the occurrence and development of RA. The dexamethasone administration group and the NF administration group may alleviate and inhibit the onset of arthritis by reducing the levels of pro-inflammatory cytokines. Serum levels of TNF-α and IL-6 were significantly increased in the model group, indicating the existence of inflammatory response during the immune response and tissue damage of RA, while the dexamethasone and NF administration groups could treat RA by reducing serum levels of TNF-α and IL-6 (Fig. 4). The mechanism was probablely that they could alleviate joint synovitis and cartilage destruction by reducing the levels of cytokines. In addition, they could also inhibit the secretion of protease by synovial cells to reduce joint damage and cartilage destruction. According to the CIA model screening, NF is the most effective fraction of *C. longifolia* in the treatment of RA.

### 3.2 Anti-RA mechanism of effective fraction

**Fig. 5:**
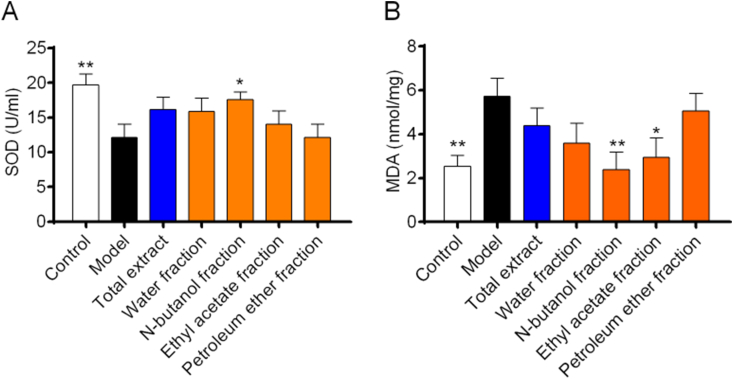
Effects of different fractions on SOD and MDA levels.

**Fig. 6:**
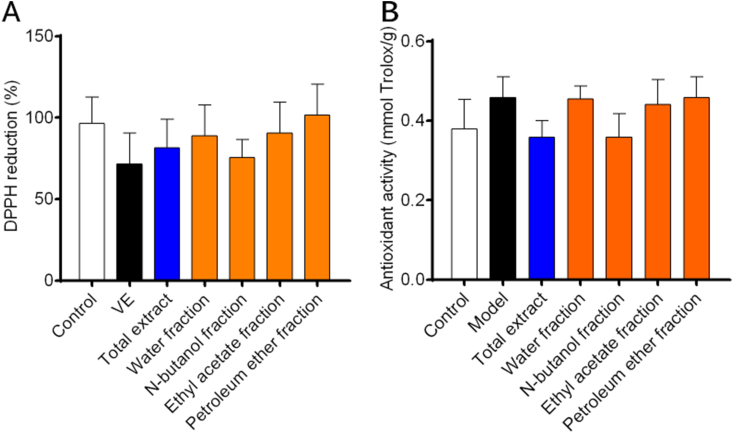
DPPH radical scavenging and antioxidant activities of different fractions.A:DPPH reduction,B:antioxidant activity.

**Fig. 7:**
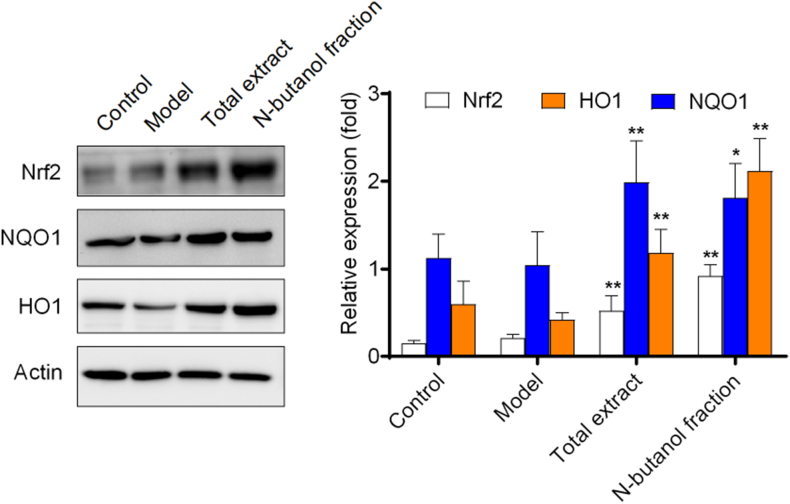
Effects of TE and NF on Nrf2, HO1 and NQO1 proteins.

**Fig. 8:**
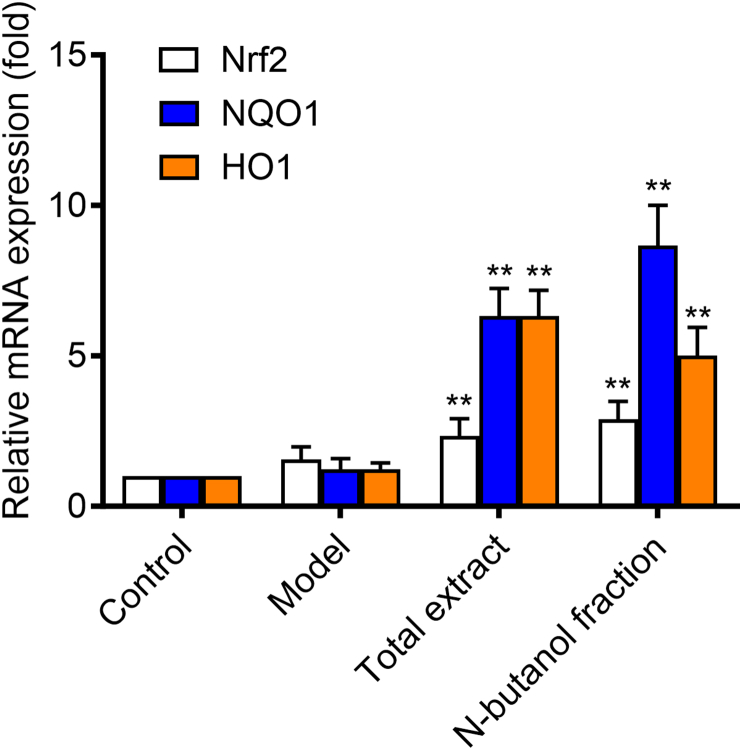
Effect of TE and NF on mRNA levels of Nrf2, HO1 and NQO1.

Nuclear factor-erythroid 2 related factor 2 (Nrf2) is a key protein in regulating the endogenous antioxidant system [22]. Nrf2 protein is degraded by programmed ubiquitination through its interaction with Keapl protein to maintain a low expression level under normal physiological conditions [23]. Transcriptional activation of Nrf2 signaling pathway could induce the expression of heme oxygenase 1 (HO1), NAD(P)H: quinone oxidoreductase (NADPH: quinone oxidoreductase l, NQO1), superoxide dismutase (SOD), and so on [[24], [25], [26]]. Activation of the Nrf2 signaling pathway could also improve the expression level of downstream antioxidant enzymes significantly, thereby enhancing the ability of the body to resist oxidative stress injuries, which had become a therapeutic approach for a variety of chronic diseases [27].As shown in Fig. 5, SOD（Fig. 5A） level was increased and MDA（Fig. 5B） level was decreased significantly in the NF administration group, suggesting that NF may have a therapeutic effect on RA by reducing the level of oxidative stress. In addition, the TE administration group and the NF administration group showed an antioxidant trend, there is no significant antioxidant activity observed at these doses in statistics.(Fig. 6). By studying the signaling pathways associated with RA, it was found that the protein expression levels of Nrf2, HO1 and NQO1 were upregulated after TE or NF administration, which indicated that the anti-arthritic effect may be related to the Nrf2 signaling pathway and the downstream HO1 and NQO1 proteins (Fig. 7). qPCR experimental results showed that the mRNA expression levels of Nrf2, HO1 and NQO1 were upregulated after TE or NF administrations as well, which confirmed that the anti-arthritic effect may be related to the Nrf2 signaling pathway and the downstream proteins HO1 and NQO1 (Fig. 8).

## 4 Conclusions

In this study, CIA rat model induced by type CII collagen was successfully constructed, the histopathological examinations showed inflammatory cell infiltration and joint injury. Therapeutic effects of TE, WF, NF, EF and PF on RA were screened. Results showed that serum levels of IL-6 and TNF-α in the model group were obviously increased compared with control group (*P* < 0.01). The serum levels of IL-6 and TNF-α in the TE administration group and the NF administration group were significantly decreased compared with the model group (*P* < 0.01). The histopathological injuries were improved. The above results indicated that *C. longifolia* had anti-RA effect, and NF was the most effective fraction, which was supported by the test of oxidative stress-related enzyme activity. These results are consistent with the results of our previous in vitro study [10]. The anti-RA effects mechanism is that the mRNA and protein expression levels of Nrf2, HO1 and NQO1 are upregulated after TE and NF administration. Therefore, the anti-RA effects mechanism may be related to the Nrf2 signaling pathway.

## Funding statement

This study was supported by the 10.13039/501100001809Natural Science Foundation of China (Grant No. 81860739) and 10.13039/501100004479Natural Science Foundation of Jiangxi Province (Grant No. 2020BABL216068).

## Data availability statement

Data will be made available on request.

## CRediT authorship contribution statement

**Yan-ping Gao:** Funding acquisition, Conceptualization. **Qiuting Ma:** Software, Data curation. **Jian Liang:** Methodology. **Qiang Wu:** Data curation, Conceptualization. **Yu-ye Zhu:** Writing – original draft, Software. **Xi-de Ye:** Writing – original draft, Investigation. **Zhiyong Liu:** Writing – review & editing, Supervision, Project administration, Conceptualization.

## Declaration of competing interest

The authors declare that they have no known competing financial interests or personal relationships that could have appeared to influence the work reported in this paper.

## Figures and Tables

**figs1:**
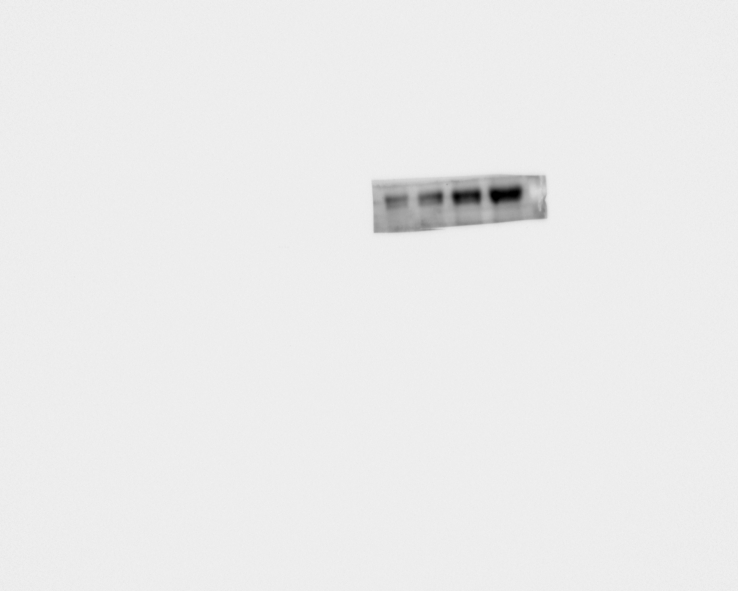


**figs2:**
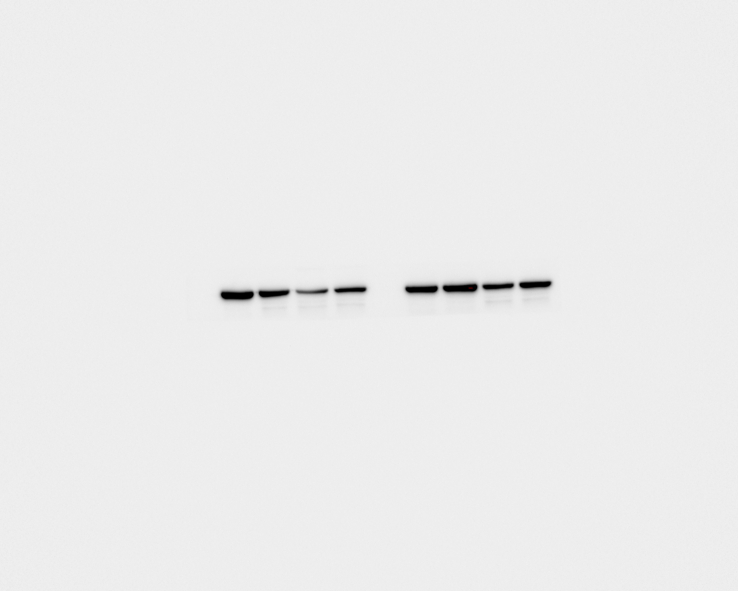


**figs3:**